# Expression Level of Transcription Factor *ART1* Is Responsible for Differential Aluminum Tolerance in *Indica* Rice

**DOI:** 10.3390/plants10040634

**Published:** 2021-03-26

**Authors:** Li Ming Sun, Jing Che, Jian Feng Ma, Ren Fang Shen

**Affiliations:** 1 State Key Laboratory of Soil and Sustainable Agriculture, Institute of Soil Science, Chinese Academy of Sciences, Nanjing 210008, China; lmsun@issas.ac.cn (L.M.S.); jche@issas.ac.cn (J.C.); 2 University of Chinese Academy of Sciences, Beijing 100049, China; 3 Institute of Plant Science and Resources, Okayama University, Chuo 2-20-1, Kurashiki 710-0046, Japan; maj@rib.okayama-u.ac.jp

**Keywords:** Al tolerance, ART1, *indica* rice, genotypic difference, transcription factor, transcriptome analysis

## Abstract

Rice is the most aluminum (Al)-tolerant species among the small grain cereals, but there are great variations in the Al tolerance between subspecies, with higher tolerance in *japonica* subspecies than *indica* subspecies. Here, we performed a screening of Al tolerance using 65 *indica* cultivars and found that there was also a large genotypic difference in Al tolerance among *indica* subspecies. Further characterization of two cultivars contrasting in Al tolerance showed that the expression level of *ART1 (ALUMINUM RESISTANCE TRANSCRIPTION FACTOR 1)* encoding a C2H2-type Zn-finger transcription factor, was higher in an Al-tolerant *indica* cultivar, Jinguoyin, than in an Al-sensitive *indica* cultivar, Kasalath. Furthermore, a dose-response experiment showed that *ART1* expression was not induced by Al in both cultivars, but Jinguoyin always showed 5.9 to 11.4-fold higher expression compared with Kasalath, irrespectively of Al concentrations. Among genes regulated by ART1, 19 genes showed higher expression in Jinguoyin than in Kasalath. This is associated with less Al accumulation in the root tip cell wall in Jinguoyin. Sequence comparison of the 2-kb promoter region of *ART1* revealed the extensive sequence polymorphism between two cultivars. Whole transcriptome analysis with RNA-seq revealed that more genes were up- and downregulated by Al in Kasalath than in Jinguoyin. Taken together, our results suggest that there is a large genotypic variation in Al tolerance in *indica* rice and that the different expression level of *ART1* is responsible for the genotypic difference in the Al tolerance.

## 1. Introduction

The toxicity of aluminum ion (mainly Al^3+^) is a major limiting factor of crop production in acid soils, which comprises around 30–40% of arable land in the world [1]. Ionic Al rapidly inhibits root elongation and uptake of water and nutrients [2,3], resulting in increased sensitivity to environmental stresses and reduced crop yields [3]. However, there are great variations in Al tolerance between plant species and cultivars within a species [4,5].

Among small grain cereals such as rice, maize, wheat, barley, and sorghum, rice (*Oryza sativa*) showed the highest Al tolerance [5,6]. Studies mainly on *japonica* cultivars showed that high Al tolerance in rice is achieved by the pyramiding of multiple mechanisms conferred by multiple genes [4,5]. These genes are involved in both the external and internal detoxification of Al and are regulated by ART1 (ALUMINUM RESISTANCE TRANSCRIPTION FACTOR 1). ART1 is a C2H2-type zinc finger transcription factor [7], which regulates at least 32 genes by binding to the core cis-acting element [GGN(T/g/a/C)V(C/A/g)S(C/G)] in the promoter region of these genes [8]. Some of these downstream genes have been functionally characterized. For example, *OsSTAR1* and *OsSTAR2 (SENSITIVE TO ALUMINUM RHIZOTOXICITY 1* and *2)* together encoded a bacterial-type ABC transporter transporting UDP-glucose, which may have modified the cell wall, resulting in decreased Al accumulation in the cell wall [9]. *OsFRDL4 (FERRIC REDUCTASE DEFECTIVE LIKE 4)* encoded a transporter responsible for the secretion of citrate from the roots to chelate Al in the rhizosphere in response to Al [10]. *OsNrat1 (NRAMP ALUMINUM TRANSPOTOER 1)* encoded a transporter located on a plasma membrane to take up trivalent Al into the cell [11], which was subsequently sequestered by OsALS1 (ALUMINUM SENSITIVE 1), a tonoplast-localized half-size ABC transporter [12]. Moreover, a plasma membrane-localized Mg transporter OsMGT1 (MAGNESIUM TRANSPORTER 1) was also required for Al-tolerance by increasing Mg uptake under Al-stressed conditions [13], and OsCDT3 (CADMIUM TOLERANT 3), a small cysteine-rich peptide with binding activity with Al, may have prevented Al from entering into the root cells [14]. The expression of *ART1* was not induced by Al [7], but ART1-regulated genes were rapidly induced by Al in the roots [7]. The knockout of these ART1-regulated genes resulted in increased Al sensitivity with different extents. Recently, a homolog of ART1 and ART2 (ALUMINUM RESISTANCE TRANSCRIPTION FACTOR 2) was also reported to be involved in Al tolerance in rice, although its contribution to Al tolerance is smaller than that of ART1 [15].

On the other hand, there is also a wide variation of Al tolerance among different subpopulations of rice; the relative degree of Al tolerance follows the following order: temperate *japonica* > tropical *japonica* > *aromatic* > *indica* = *aus* [5]. Several studies showed that these differences in Al tolerance between rice subspecies are partially attributed to the differential expression of ART1-regulated genes. For example, the expression level of *Nrat1* and *OsFRDL4* was higher in *japonica* subspecies than in *indica* subspecies [16,17]. Furthermore, a 1.2-kb insertion in the promoter region of *OsFRDL4* in *japonica* subspecies, but not in the *indica* or wild rice subspecies, was responsible for Al tolerance by enhancing the expression of *OsFRDL4* [18]. In addition to the subspecies difference, there is also a genotypic difference in Al tolerance within the same subspecies [5]. However, the mechanisms responsible for the genotypic difference in Al tolerance are poorly understood, especially in the *indica* subspecies. In the present study, we first investigated the genotypic difference in *indica* subspecies by screening 65 cultivars from the world core collection. We selected two *indica* cultivars with contrasting Al tolerance for further physiological and molecular characterization and found that the expression level of *ART1* is probably associated with genotypic differences in Al tolerance in *indica* subspecies.

## 2. Materials and Methods

### 2.1. Screening of Al Tolerance in Indica Cultivars

To compare Al tolerance in *indica* cultivars, we performed a screening using 65 *indica* cultivars selected from the world and Japan rice core collections (Supplementary Figure S1). The seeds were soaked in water at 30 °C for 2 days and then placed on a net floated on a 0.5 mM CaCl_2_ solution in a 20-L plastic container. After 3–4 days, the seedlings were exposed to a 0.5 mM CaCl_2_ solution (pH 4.5) with or without 50 μM Al. Before and after 24 h exposure, the root length was measured using a ruler. The relative root elongation was calculated as the following: root elongation with Al/root elongation without Al ×100. There were 5 to 10 replicates made for each cultivar.

### 2.2. Al Tolerance Evaluation in Hydroponic Solution

Based on the screening results, we selected two cultivars, Kasalath and Jinguoyin, for further analysis. We first compared their Al tolerance at different Al concentrations by exposing the 4-day-old seedlings to a 0.5 mM CaCl_2_ solution (pH 4.5) containing 0, 30, 50, and 100 μM Al in a 1.5-L container for 24 h. The root lengths were measured before and after the treatment, as described above, with 10 replicates. A time-course experiment was also conducted by exposing the 4-day-old seedlings to a 0.5 mM CaCl_2_ (pH 4.5) solution containing 0 or 50 μM Al. At different time points, indicated in Figure 2, the root lengths were measured.

### 2.3. Growth Test in Acid and Neutral Soils

For the soil test, seeds of both Kasalath and Jinguoyin were surface-sterilized for 30 min in a 10% (*v*/*v*) H_2_O_2_ solution, washed thoroughly with deionized water, soaked in deionized water at 30 °C overnight, and germinated in darkness for 2 days. The germinated seeds were transferred to acid soil (Quaternary red clay soil, 0–20 cm, pH 4.3, which was collected from a pine forest area in Yingtan, Jiangxi, China) or neutral soil (pH 7.0), amended with CaCO_3_ at 8.0 g (kg soil)^−1^. Pictures were taken after 4 days, and the root lengths were measured with a ruler.

### 2.4. Determination of Al Accumulation in the Roots

For the determination of Al content in the root tips, the 4-day-old seedlings were exposed to a 0.5 mM CaCl_2_ (pH 4.5) solution containing 50 μM Al for 24 h. After the roots were washed with 0.5 mM CaCl_2_ three times, the root segments (0–1 cm from the root tip) were excised and placed in a 1.5 mL plastic tube containing 1 mL of 2 N HCl for at least 24 h with occasional vortex. For the collection of the root cell sap, the excised root segments were placed on a filter in a tube and frozen at −80 ℃ overnight. After rapid thawing at room temperature, the cell sap was collected by centrifugation at 20,600 *g* for 10 min. The pellet was washed three times (5 min for each washing) with 70% ethanol to remove membrane fractions, and then the Al in the cell wall fraction was extracted using 2 M HCl. The Al concentration in the extraction solution was determined by Inductively coupled plasma mass spectrometry using an Agilent 7700 mass spectrometer (Palo Alto, Santa Clara, CA, USA). Three replicates were made for each treatment.

### 2.5. RNA Sequencing Analysis

For RNA-seq analysis, 4-day-old seedlings of the Kasalath and Jinguoyin were exposed to 0 or 50 μM Al in a 0.5 mM CaCl_2_ solution (pH4.5). After 6 h, the root tips (0–1 cm) were excised with a razor, with four replicates, and immediately frozen in liquid nitrogen. The RNA was extracted using the TIANGEN TGuide Plant RNA Kit (Beijing, China). After RNA quantification and qualification, a total amount of 1 μg RNA per sample was used as the input material for library construction. The sequencing libraries were generated using NEBNext UltraTM RNA Library Prep Kit for Illumina (Ipswich, UK), following the manufacturer’s recommendations, and index codes were added to attribute sequences to each sample. Then the cDNA libraries were subjected to paired-end sequencing using an Illumina HiSeq 2500 system (San Diego, CA, USA). The raw sequencing data were filtered using a Fastx toolkit (v 0.0.14–1) to get high-quality clean data, which was then mapped to the reference Nipponbare rice genome (http://rice.plantbiology.msu.edu) using Hisat2 (Version 2.1.0) [19]. A transcript assembly, a calculation of the normalized transcript abundance (reads per kilobase million), was performed using StringTie (v2.0.4) [20]. Differential expression analysis of two conditions/groups was performed using the DEseq2 (Version 1.42.0) [21]. Genes with an adjusted *p*-value < 0.05 and a fold change ≥2 were assigned as differentially expressed. Gene Ontology (GO) enrichment analysis of the differentially expressed genes (DEGs) was implemented by the GOseq R packages (Version 1.42.0) [22] based on Wallenius non-central hyper-geometric distribution. The KOBAS software (Version 3.0.3) [23] was used to test the statistical enrichment of the DEGs in the Kyoto Encyclopedia of Genes and Genomes (KEGG) pathways.

### 2.6. Quantitative Real-Time PCR (qRT-PCR) Analysis

To investigate gene expression, 4-day-old seedlings of Kasalath and Jinguoyin were exposed to a 0.5 mM CaCl_2_ (pH 4.5) solution containing 0 or 50 μM Al for 24 h, then root tips (0–1 cm) were excised and frozen in liquid nitrogen immediately. For a dose-response experiment on *ART1* expression, 4-day-old seedlings of Kasalath and Jinguoyin were exposed to a 0.5 mM CaCl_2_ (pH 4.5) solution containing 0, 30, 50, and 100 μM Al for 24 h. The RNA was extracted as described before and converted to cDNA using the Takara PrimeScript RT reagent kit (Kyoto, Japan) following the manufacturer’s instructions. For quantitative real-time PCR analysis, 1 µL 10-fold-diluted cDNA was used for the quantitative analysis of gene expression performed with a Takara SYBR Premix ExTaq (Kyoto, Japan), with the pairs of gene-specific primers. All primer sequences employed for the qRT-PCR have been listed in Supplementary Table S1. *Histone H3* was used as an internal control. The relative gene expression levels were calculated by the comparative C_t_ method. Three independent biological replicates were made for each treatment.

### 2.7. Sequence Comparison of the Promoter and Coding Sequence Regions of ART1 

The genome DNA was isolated using the hexadecyl trimethyl ammonium bromide (CATB) method from fresh leaves of Kasalath and Jinguoyin. Then, sequencing libraries were generated using a RsaIof restriction enzyme and subjected to paired-end sequencing using an Illumina HiSeq 2500 system (San Diego, CA, USA). The raw sequencing data were filtered using a Fastx toolkit to get high-quality clean data, which was then mapped to the reference Nipponbare rice genome (http://rice.plantbiology.msu.edu) using BWA-MEM (0.7.10-r789) [24]. Variant calling was conducted using GATK Tools (version 3.6) [25]. The sequence differences in the promoter and coding region of *ART1* were listed.

### 2.8. Statistical Analysis

Student’s *t*-test and Tukey’s test were applied to test differences among treatments at *p*-values < 0.05.

## 3. Results

### 3.1. Screening of Al Tolerance in Indica Cultivars

To evaluate Al tolerance in 65 *indica* cultivars, we compared the relative root elongation (RRE) under Al stress conditions. The RRE ranged from 15.9% to 74.6%, indicating that there was a genotypic difference in Al tolerance in *indica* subspecies (Supplementary Figure S1). For further physiological and molecular analysis, we selected Jinguoyin and Kasalath, which showed similar root growth under −Al conditions, but contrasted in Al tolerance (Supplementary Figure S1).

### 3.2. Al Tolerance Test of Two Indica Cultivars

**Figure 1:**
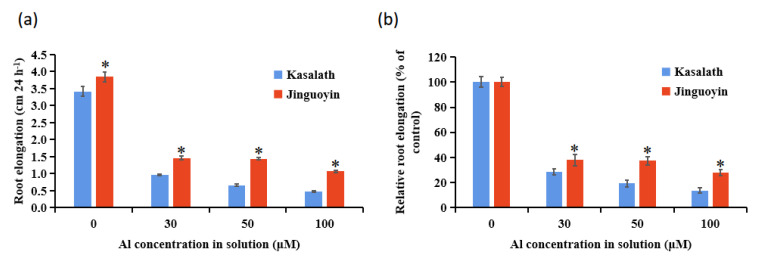
Al-dependent root elongation in two indica rice cultivars. (**a**) Absolute root elongation; (**b**) relative root elongation (RRE). Four-day-old seedlings of both Kasalath and Jinguoyin were exposed to a 0.5 mM CaCl_2_ (pH 4.5) solution containing 0, 30, 50, or 100 μM Al for 24 h. Root lengths were measured with a ruler before and after treatment and RRE was calculated as the ratio of root elongation with Al to root elongation without Al. Data are mean ± SD (n = 10). Asterisks (*) indicate significant differences between Kasalath and Jinguoyin at each Al concentration at *p*-values <0.05 by the Student’s *t*-test.

**Figure 2:**
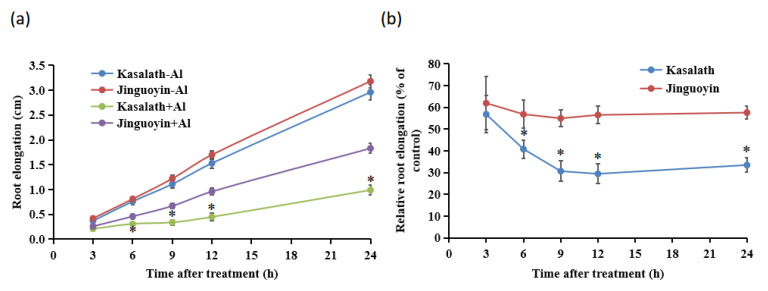
Time-dependent root elongation in two *indica* rice cultivars with and without Al. Absolute root elongation (**a**); relative root elongation (**b**). Four-day-old seedlings of both Kasalath and Jinguoyin were exposed to a 0.5 mM CaCl_2_ (pH 4.5) solution containing 0 or 50 μM Al for up to 24 h. Root lengths were measured with a ruler at different time points. Data are mean ± SD (n = 10). Asterisks (*) indicate significant differences between Kasalath and Jinguoyin in the presence of Al at each time point at *p*-values <0.05 by the Student’s *t*-test.

A dose-response experiment showed that the root elongation was inhibited by Al more in Kasalath than in Jinguoyin at all Al concentrations tested (Figure 1). The RRE of Kasalath was 71.6%, 80.7%, and 86.3%, respectively, after exposure to 30, 50, and 100 μM Al for 24 h, whereas that of Jinguoyin was 62.1%, 62.6%, and 72.2%, respectively (Figure 1b). In the time-course experiment, there was no difference in Al-inhibited root elongation between Jinguoyin and Kasalath at 3 h after Al exposure (Figure 2a). However, at 6 h and thereafter, the RRE was significantly higher in Jinguoyin than in Kasalath (Figure 2b). These results indicate that Jinguoyin was more tolerant to Al than Kasalath, supporting our screening results (Supplementary Figure S1).

**Figure 3:**
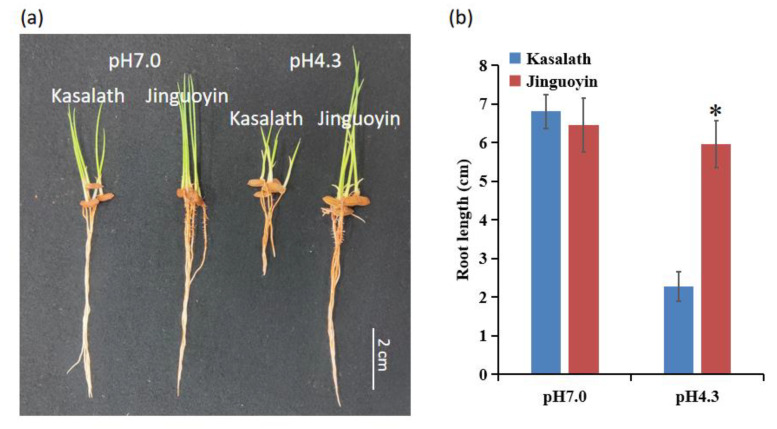
Root growth in acid and neutral soils. Root growth phenotype of two cultivars grown in acid and neutral soil (**a**); root length (**b**). Germinated seeds of Kasalath and Jinguoyin were sowed in acid soil with a pH of 4.3, or neutral soil with a pH of 7.0, and grown for 4 d. Neutral soil is modified from acid soil by amending it with CaCO_3_ at 8.0 g (kg soil)^−1^. Data are mean ± SD (n = 5). Asterisks (*) indicate significant differences between Kasalath and Jinguoyin at *p*-values of <0.05 by the Student’s *t*-test.

To confirm the Al tolerance in two cultivars, the root growth was also compared when grown in acid soil and neutral soil. In neutral soil (pH 7.0), the root growths of Kasalath and Jinguoyin were similar (Figure 3). However, in acid soil (pH 4.3), the root growth of Kasalath was largely inhibited compared with that in neutral soil, while the root growth of Jinguoyin was hardly affected (Figure 3). Consistently, the root length of Jinguoyin was longer than Kasalath in acid soil, while no difference was observed in neutral soil (Figure 3). These results confirm that Jinguoyin is an Al-tolerant *indica* cultivar, while Kasalath is an Al-sensitive *indica* cultivar.

### 3.3. Al Accumulation in the Root Tips

**Figure 4:**
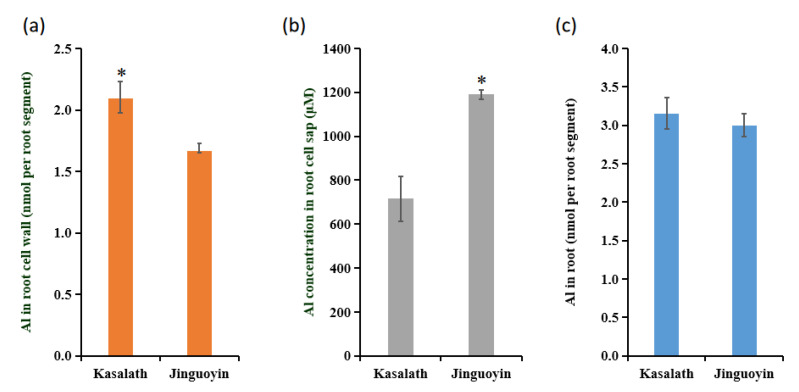
Al accumulation in the root tips. (**a**) Root tip cell wall Al content; (**b**) root tip cell sap Al concentration, and (**c**) total root tip Al content of two *indica* cultivars. Four-day-old seedlings were exposed to a 0.5 mM CaCl_2_ (pH 4.5) solution containing 50 μM Al for 24 h. Then, the root tips (0–1 cm) were excised and the cell wall and cell sap were fractioned. Al concentration was determined by Inductively coupled plasma mass spectrometry. Data are mean ± SD (n = 3). Asterisks (*) indicate significant differences between Kasalath and Jinguoyin at *p*-values of <0.05 by the Student’s *t*-test.

We compared Al accumulation in the root tips (0–1 cm from the apex), in the root cell wall, and root cell sap between two cultivars. The total Al content in the root tip showed no difference between Kasalath and Jinguoyin (Figure 4a). However, Kasalath accumulated significantly higher levels of Al in the cell wall, but lower levels of Al in the cell sap than Jinguoyin in the root tips (Figure 4b,c). These results indicate that the Al partition between the cell wall and inside the cells in the root tips differed between Kasalath and Jinguoyin.

### 3.4. Differentially Expressed Genes (DEGs) between Kasalath and Jinguoyin

**Figure 5:**
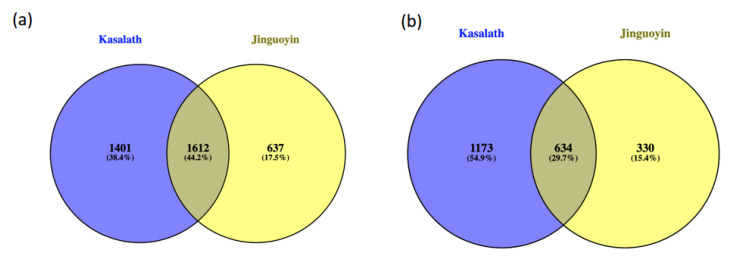
Numbers of differential expressed genes induced by Al. Genes up- (**a**) and downregulated (**b**) by Al in Kasalath and Jinguoyin. Four-day-old seedlings were exposed to a 0.5 mM CaCl_2_ (pH 4.5) solution containing 0 or 50 μM Al. After 6 h, the root tips (0–1 cm) were excited and subjected to RNA-seq analysis. Genes where the expression is significantly higher than two folds are shown (adjusted *p*-value of <0.05).

To identify the candidate genes contributing to high Al tolerance in Jinguoyin, we compared the transcriptomic profiles of genes in the root tips between Kasalath and Jinguoyin by RNA sequencing. The RNA sequencing analysis showed that 3013 and 2249 genes, including 1612 common genes (greater than twofold), were upregulated by Al in Kasalath and Jinguoyin, respectively, after exposure to Al for 6 h (Figure 5a). On the other hand, 1807 and 964 genes, including 634 common genes (higher than twofold), were downregulated by Al in Kasalath and Jinguoyin, respectively (Figure 5b).

### 3.5. Verification of RNA-seq Results by Quantitative Real-Time PCR

To validate the reliability of the RNA sequencing data, we randomly selected 17 genes for the quantitative real-time PCR (qRT-PCR) analysis. There was a good correlation (r = 0.92) between the RNA-seq data and the qRT-PCR results (Supplementary Figure S2).

### 3.6. GO and KEGG Pathway Enrichment Analysis

**Figure 6:**
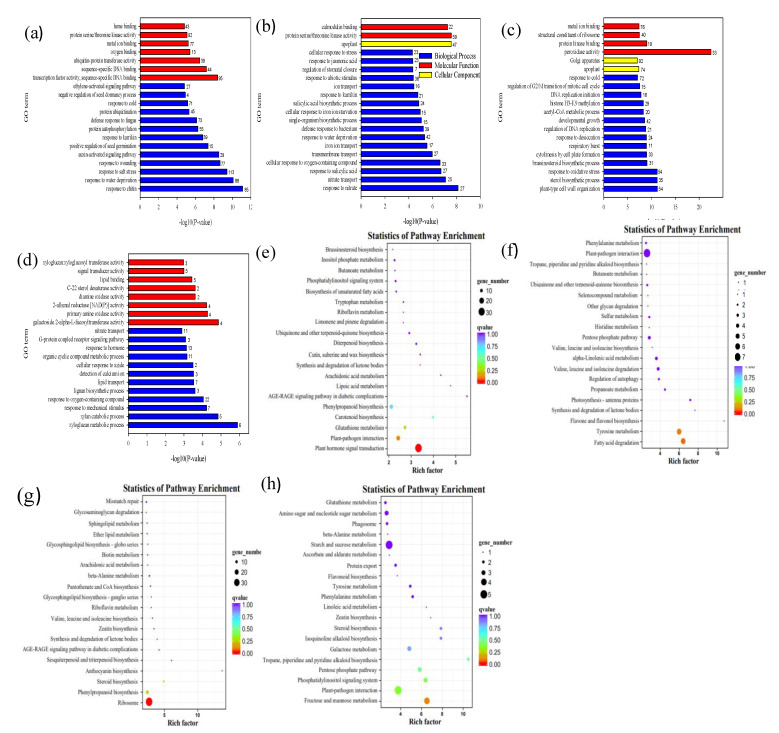
Functional analysis of differential expressed genes induced by Al in two indica rice cultivars. Gene Ontology (GO) enrichment analysis (**a**–**d**); Kyoto Encyclopedia of Genes and Genomes (KEGG) pathway enrichment analysis (**e**–**h**) of uniquely upregulated genes (**a**,**b**,**e**,**f**) and downregulated (**c**,**d**,**g**,**h**) genes in Kasalath (**a**,**c**,**e**,**g**) and Jinguoyin (**b**,**d**,**f**,**h**). The 20 most enriched GO terms with the lowest *p*-values are listed.

Among the up- and downregulated genes, we performed GO and KEGG pathway enrichment analysis using genes differentially expressed in Jinguoyin and Kasalath. Among 637 up-regulated DEGs in Jinguoyin, “response to nitrate”, “protein serine/threonine kinase activity”, and “apoplast” were the most enriched GO terms, while “response to chitin”, “response to water deprivation”, and “response to salt stress” were the most enriched GO terms in the 1401 upregulated DEGs of Kasalath (Figure 6a,b). There were no significantly enriched KEGG pathways in the upregulated DEGs in Jinguoyin, while “plant hormone signal transduction” was the only significantly enriched KEGG pathway in the upregulated genes of Kasalath (Figure 6e,f).

Among the 1173 downregulated DEGs in Kasalath, “peroxidase activity”, “plant-type cell wall organization”, and “sterol biosynthetic process” are the most enriched GO terms, while “xyloglucan metabolic process”, “galactoside 2-alpha-L-fucosyltransferase activity”, and “xylan catabolic process” were the most enriched GO terms in the 330 up-regulated DEGs of Jinguoyin (Figure 6c,d). In addition, “ribosome” was a uniquely enriched KEGG pathway in the downregulated DEGs of Kasalath, while there were no significantly enriched KEGG pathways in the upregulated DEGs in Jinguoyin (Figure 6g,h). Impaired function of “peroxidase activity” and “ribosome” may be the cause of low Al tolerance in Kasalath.

### 3.7. Expression Profile of ART1-Regulated Genes in Kasalath and Jinguoyin

**Figure 7:**
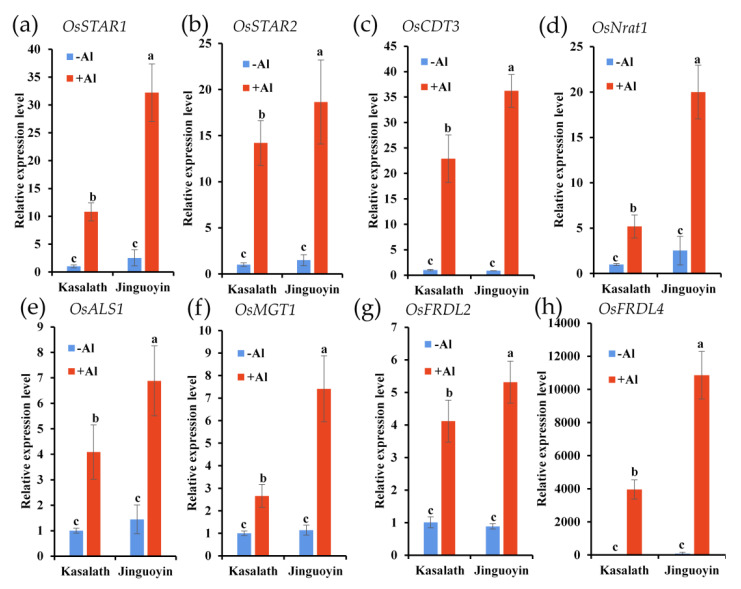
Gene expression pattern analysis of ART1-regulated genes in two *indica* rice cultivars. The expression level of *OsSTAR1* (**a**), *OsSTAR2* (**b**), *OsCDT3* (**c**), *OsNrat1* (**d**), *OsALS1* (**e**), *OsMGT1* (**f**), *OsFRDL2* (**g**), and *OsFRDL4* (**h**) was determined by quantitative RT-PCR. *Histone H3* was used as an internal standard. Expression level relative to Kasalath root (−Al) is shown. Seedlings of Kasalath and Jinguoyin were exposed to a 0.5 mM CaCl_2_ (pH 4.5) solution containing 0 or 50 μM Al for 24 h. Data are mean ± SD (n = 3). Different letters indicate significant differences at a *p*-value <0.05 by Tukey’s test.

Since ART1 is a key regulator of Al tolerance in *japonica* cultivars, we extracted the expression profile of ART1-regulated genes from RNA-seq data. Among the 32 genes regulated by ART1 [8,16], 23 genes were upregulated by Al in both Kasalath and Jinguoyin (Table 1), but 18 genes were upregulated more in Jinguoyin than in Kasalath. These results were confirmed by qRT-PCR for some ART1-regulated genes, including *OsSTAR1*, *OsSTAR2*, *OsCDT3*, *OsNrat1*, *OsALS1*, *OsMGT1*, *OsFRDL2*, and *OsFRDL4* (Figure 7a–h). 

**Figure 8:**
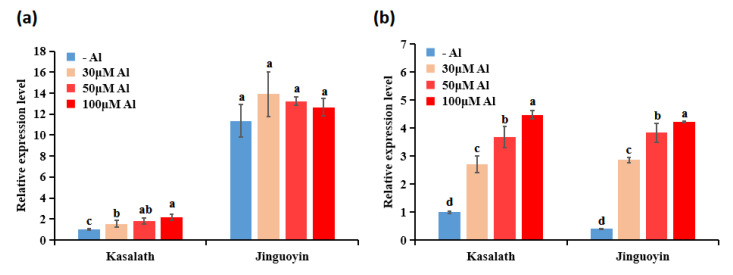
Dose-dependent expression of *ART1* (**a**) and *ART2* (**b**) in rice roots of Kasalath and Jinguoyin. 4-day-old seedlings were exposed to a 0.5 mM CaCl_2_ (pH 4.5) solution containing 0, 30, 50, and 100 μM Al for 24 h. Data are mean ± SD (n = 3). Different letters indicate significant differences between different treatments in Kasalath and Jinguoyin at *p*-values <0.05 by Tukey’s test.

We then compared the expression of *ART1* in two cultivars. As a result, the expression level of *ART1* was higher in Jinguoyin than in Kasalath, both in the absence and presence of Al (Figure 8a). Furthermore, the dose-response experiment showed that the *ART1* expression was constitutively higher in Jinguoyin than in Kasalath independent of Al concentrations (Figure 8a). On the other hand, no difference in the *ART2* expression was found (Figure 8b).

### 3.8. Sequence Comparison of Promoter and Coding Sequence Region of ART1

To investigate the mechanism for different expression levels of *ART1* between Kasalath and Jinguoyin, we compared the sequence of the promoter (2.0 kb) and coding sequencing region of *ART1*. The results showed that compared to the *japonica* rice reference genome (Nipponbare), the promoter and coding sequence region of Jinguoyin was exactly the same as Nipponbare (Supplementary Figure S3). However, there were 14 single nucleotide polymorphisms (SNPs) and 3 insertion-deletions (Indels) in the promoter (Supplementary Figure S3a), and 10 SNPs and 3 Indels in the coding sequence region of Kasalath compared with Jinguoyin (Supplementary Figure S3b).

## 4. Discussion

There are two major subspecies in rice, including *japonica* rice and *indica* rice. *Indica* rice is usually cultivated in tropics and subtropics, where acid soils are widely distributed. However, its tolerance to Al toxicity is generally lower than *japonica* rice [5]. In the present study, we found that there was also a wide genotypic difference in Al tolerance in *indica* rice based on the screening of 65 *indica* cultivars from the world and Japan rice core collections (Supplementary Figure S1). Further characterization of two *indica* cultivars contrasting in Al tolerance suggests that the expression level of *ART1* is responsible for the genotypic difference in Al tolerance.

ART1 is a key transcription factor for Al resistance identified in rice [7]. It regulates at least 32 genes involved in detoxifying Al both externally and internally [7,9,10,11,12,13,14,18]. The expression level of *ART1* in Al-tolerant Jinguoyin was 5.9 to 11.4-fold higher than in Al-sensitive Kasalath, independently of Al concentrations (Fig. 8a). This higher expression of *ART1* in Jinguoyin resulted in the enhanced expression of downstream genes, including *OsSTAR1*, *OsSTAR2*, *OsCDT3*, *OsNrat1*, *OsALS1*, *OsMGT1*, *OsFRDL2*, and *OsFRDL4* (Table 1, Figure 7), subsequently contributing to higher Al tolerance in Jinguoyin. This is partially supported by the analysis of the cell wall Al. Al binding to the cell wall decreases the plasticity and inhibits root elongation [26]. Al-tolerant Jinguoyin showed less Al accumulation in the cell wall of the root tip compared with Kasalath (Figure 4). This may be attributed to the higher *Nrat1* expression in Jinguoyin (Figure 7d). Nrat1 is a plasma-localized transporter for Al^3+^, which is required for further sequestration of Al into the vacuoles by OsALS1 [11,12]. The knockout of this gene also resulted in increased Al accumulation in the root cell wall [11]. Although the mechanism responsible for higher *ART1* expression in Jinguoyin is unknown, the difference in the sequence of the *ART1* promoter may be involved in regulating *ART1* expression (Supplementary Material, Figure S3).

Previously, *ART1* was identified as a gene responsible for the QTL Alt2.1 on chromosome 12 in a population derived from a cross between Azucena (Al-tolerant, *japonica* rice) and IR64 (Al-sensitive, *indica* rice) [27]. This explains a large proportion (>19%) of the variation in Al tolerance. However, different from our study, there was no difference in the expression level of *ART1* in the two cultivars [20]. The sequence polymorphism in the coding region may be responsible for a different Al response through affecting protein folding and interactions with the target gene promoter. It was reported that compared to Al-sensitive rice IR 1552, Al-tolerant rice ARR09 contained an SNP in the coding sequence region of *OsNrat1* [28]. In addition to the regulation of *ART1* itself, several reports also showed that *cis*-element numbers were altered in the promoter region of downstream genes of ART1. For example, a higher Al tolerance in *Holcus lanatus* is achieved by increased numbers of *cis*-acting elements regulating the expression of *ALMT1* involved in the Al-induced secretion of malate [29]. A 1.2 kb re-transposon insertion in the promoter region of *OsFRDL4* increased *cis*-acting element numbers, resulting in a higher expression of *OsFRDL4* [18]. These findings indicate that the regulation mechanism of Al-tolerance differs with plant species and cultivars at a transcriptional level.

Recently, ART2, a homolog of ART1, was also involved in Al tolerance in rice [15]. Unlike *ART1*, the expression of *ART2* was induced by Al. In the present study, we found that the expression of *ART2* was induced by Al in both cultivars (Figure 8b). However, there was no difference in the expression level between the two cultivars, indicating that *ART2* is not involved in differential Al tolerance between Jinguoyin and Kasalath.

Transcriptome analysis revealed that more genes were up- and downregulated by Al in Al-sensitive Kasalath compared with Al-tolerant Jinguoyin (Figure 5). This may be attributed to the higher Al sensitivity of Kasalath, resulting in the induction of more genes caused by Al toxicity. GO enrichment analysis showed that these genes have different biological functions (Figure 6a–h). For example, “peroxidase activity” was one of the most enriched GO terms in the downregulated DEGs in Kasalath, as Al can cause oxidative stress, and therefore, impaired peroxidase activity reduced its adaptability to Al stress. However, it remains to be investigated whether these DEGs are also involved in differential Al tolerance in addition to *ART1* expression in *indica* rice.

In conclusion, we found a large genotypic difference in Al tolerance in *indica* rice. Furthermore, we found that the different expression of *ART1* may be responsible for the genotypic Al tolerance in *indica* rice.

## Figures and Tables

**Table 1:** Expression changes of ART1-regulated genes in the root tips of Kasalath and Jinguoyin. RNA sequencing was performed with Kasalath and Jinguoyin exposed to 50 µM AlCl_3_ for 6 h with four replicates.

RAPDB ID	Kasalath			Jinguoyin			Description
	Fold Change	Q Value	Significant	Fold Change	Q Value	Significant	
	(+Al/−Al)			(+Al/−Al)			
Os01g0178300	22.56	0.00	yes	40.18	0.00	yes	OsCDT3
Os01g0652100	1.84	0.00	no	1.57	0.00	no	Protein of unknown function DUF231 domain-containing protein
Os01g0716500	4.01	0.00	yes	6.21	0.00	yes	Methyltransferase type 12 domain-containing protein
Os01g0731600	37.58	0.00	yes	10.97	0.00	yes	Similar to pathogen-related protein
Os01g0766300	7.27	0.02	yes	13.98	0.00	yes	Conserved hypothetical protein
Os01g0860500	7.15	0.00	yes	1.51	0.00	no	Chitinase
Os01g0869200	2.42	0.00	yes	5.64	0.00	yes	OsMGT1
Os01g0919100	712.05	0.00	yes	369.73	0.00	yes	OsFRDL4
Os01g0919200	38.08	0.00	yes	25.89	0.00	yes	Bacterial transferase hexapeptide repeat domain-containing protein
Os02g0131800	3.91	0.00	yes	5.67	0.00	yes	OsNramp4/OsNrat1
Os02g0186800	21.19	0.00	yes	18.89	0.00	yes	Cytochrome P450 family protein
Os02g0755900	6.29	0.00	yes	11.63	0.00	yes	UDP-glucuronosyl/UDP-glucosyltransferase domain-containing protein
Os02g0770800	32.18	0.00	yes	27.40	0.00	yes	NADH/NADPH-dependent nitrate reductase
Os03g0126900	3.42	0.00	yes	6.21	0.00	yes	Conserved hypothetical protein
Os03g0304100	inf	0.05	no	23.84	0.07	no	Conserved hypothetical protein
Os03g0755100	3.61	0.00	yes	4.89	0.00	yes	OsALS1
Os03g0760800	5.48	0.00	yes	15.68	0.00	yes	A member of the GAST (gibberellin (GA)-Stimulated Transcript) family
Os04g0494900	4.94	0.00	yes	13.39	0.00	yes	Similar to Unidentified precursor
Os04g0583500	1.66	0.00	no	1.58	0.00	no	OsEXPA10
Os05g0119000	14.14	0.11	no	0.40	0.64	no	OsSTAR2
Os06g0695800	10.46	0.00	yes	8.93	0.00	yes	OsSTAR1
Os07g0493100	5.28	0.00	yes	12.71	0.00	yes	Hypothetical protein
Os07g0587300	15.14	0.00	yes	21.31	0.00	yes	Hypothetical protein
Os09g0426800	0.82	0.36	no	2.11	0.00	yes	Homologue of WAX2/GL1, Synthesis of leaf cuticular wax
Os09g0479900	1.48	0.07	no	2.71	0.00	yes	Similar to Subtilisin-like protease
Os10g0206800	4.37	0.00	yes	6.00	0.00	yes	OsFRDL2
Os10g0524600	1.59	0.00	no	4.40	0.00	yes	Peptidase S8, subtilisin-related domain-containing protein
Os10g0578800	4.50	0.00	yes	8.03	0.00	yes	Similar to LrgB-like family protein
Os11g0490100	2.88	0.00	yes	2.41	0.00	yes	Protein of unknown function DUF579, plant family protein
Os12g0227400	9.44	0.00	yes	22.18	0.00	yes	Allyl alcohol dehydrogenase
Os04g0419100	6.34	0.00	yes	6.01	0.00	yes	Conserved hypothetical protein

## Data Availability

The data presented in this study are available on request from the corresponding author. The RNA-seq data is available on the Sequence Read Archive (SRA) of the NCBI with Accession ID PRJNA715939.

## Supplementary Materials

The following are available online at https://www.mdpi.com/article/10.3390/plants10040634/s1. Additional Supplementary Information may be found in the online version of this article. Supplementary Figure S1. Al tolerance of 65 indica rice varieties. Supplementary Figure S2. Correlation of the gene expression ratio between RNA-seq data and quantitative real-time PCR (qRT-PCR) results. Supplementary Figure S3. Sequence comparison of the promoter (a) and coding sequence (CDS) regions (b) of ART1 between Kasalath and Jinguoyin. Supplementary Table S1 Primer sequences used for investigating gene expression.

## References

[1] Von Uexkull H.R., Mutert E.W. (1995). Global extent, development and economic impact of acid soils. Plant Soil.

[2] Ma J.F. (2007). Syndrome of aluminum toxicity and diversity of aluminum resistance in higher plants. Int. Rev. Cytol..

[3] Kochian L.V., Pineros M.A., Liu J., Magalhaes J.V. (2015). Plant Adaptation to Acid Soils: The Molecular Basis for Crop Aluminum Resistance. Annu. Rev. Plant Biol..

[4] Ma J.F., Shen R., Zhao Z., Wissuwa M., Takeuchi Y., Ebitani T., Yano M. (2002). Response of rice to Al stress and identification of quantitative trait Loci for Al tolerance. Plant Cell Physiol..

[5] Famoso A.N., Zhao K., Clark R.T., Tung C.W., Wright M.H., Bustamante C., Kochian L.V., McCouch S.R. (2011). Genetic architecture of aluminum tolerance in rice (*Oryza sativa*) determined through genome-wide association analysis and QTL mapping. PLoS Genet..

[6] Ma J.F., Chen Z.C., Shen R.F. (2014). Molecular mechanisms of Al tolerance in gramineous plants. Plant Soil.

[7] Yamaji N., Huang C.F., Nagao S., Yano M., Sato Y., Nagamura Y., Ma J.F. (2009). A zinc finger transcription factor ART1 regulates multiple genes implicated in aluminum tolerance in rice. Plant Cell.

[8] Tsutsui T., Yamaji N., Ma J.F. (2011). Identification of a cis-acting element of ART1, a C2H2-type zinc-finger transcription factor for aluminum tolerance in rice. Plant Physiol..

[9] Huang C.F., Yamaji N., Mitani N., Yano M., Nagamura Y., Ma J.F. (2009). A bacterial-type ABC transporter is involved in aluminum tolerance in rice. Plant Cell.

[10] Yokosho K., Yamaji N., Ma J.F. (2011). An Al-inducible MATE gene is involved in external detoxification of Al in rice. Plant J..

[11] Xia J., Yamaji N., Kasai T., Ma J.F. (2010). Plasma membrane-localized transporter for aluminum in rice. Proc. Natl. Acad. Sci. USA.

[12] Huang C.F., Yamaji N., Chen Z., Ma J.F. (2012). A tonoplast-localized half-size ABC transporter is required for internal detoxification of aluminum in rice. Plant J..

[13] Chen Z.C., Yamaji N., Motoyama R., Nagamura Y., Ma J.F. (2012). Up-Regulation of a Magnesium Transporter Gene OsMGT1 Is Required for Conferring Aluminum Tolerance in Rice. Plant Physiol..

[14] Xia J.X., Yamaji N., Ma J.F. (2013). A plasma membrane-localized small peptide is involved in rice aluminum tolerance. Plant J..

[15] Che J., Tsutsui T., Yokosho K., Yamaji N., Ma J.F. (2018). Functional characterization of an aluminum (Al)-inducible transcription factor, ART2, revealed a different pathway for Al tolerance in rice. New Phytol..

[16] Xia J., Yamaji N., Che J., Shen R.F., Ma J.F. (2014). Differential expression of Nrat1 is responsible for Al-tolerance QTL on chromosome 2 in rice. J. Exp. Bot..

[17] Li J.Y., Liu J., Dong D., Jia X., McCouch S.R., Kochian L.V. (2014). Natural variation underlies alterations in Nramp aluminum transporter (NRAT1) expression and function that play a key role in rice aluminum tolerance. Proc. Natl. Acad. Sci. USA.

[18] Yokosho K., Yamaji N., Fujii-Kashino M., Ma J.F. (2016). Functional Analysis of a MATE Gene OsFRDL2 Revealed its Involvement in Al-Induced Secretion of Citrate, but a Lower Contribution to Al Tolerance in Rice. Plant Cell Physiol..

[19] Kim D., Langmead B., Salzberg S.L. (2015). HISAT: A fast spliced aligner with low memory requirements. Nat. Methods.

[20] Pertea M., Pertea G.M., Antonescu C.M., Chang T.C., Mendell J.T., Salzberg S.L. (2015). StringTie enables improved reconstruction of a transcriptome from RNA-seq reads. Nat. Biotechnol..

[21] Wang L.K., Feng Z.X., Wang X., Wang X.W. (2010). DEGseq: An R package for identifying differentially expressed genes from RNA-seq data. Bioinformatics.

[22] Young M.D., Wakefield M.J., Smyth G.K., Oshlack A. (2010). Gene ontology analysis for RNA-seq: Accounting for selection bias. Genome Biol..

[23] Mao X., Cai T., Olyarchuk J.G., Wei L. (2005). Automated genome annotation and pathway identification using the KEGG Orthology (KO) as a controlled vocabulary. Bioinformatics.

[24] Li H., Durbin R. (2009). Fast and accurate short read alignment with Burrows Wheeler Transform. Bioinformatics.

[25] McKenna A., Hanna M., Banks E., Sivachenko A., Cibulskis K., Kernytsky A., Garimella K., Altshuler D., Gabriel S., Daly M. (2010). The Genome Analysis Toolkit: A MapReduce framework for analyzing next-generation DNA sequencing data. Genome Res..

[26] Ma J.F., Shen R.F., Nagao S., Tanimoto E. (2004). Aluminum targets elongating cells by reducing cell wall extensibility in wheat roots. Plant Cell Physiol..

[27] Arbelaez J.D., Maron L.G., Jobe T.O., Piñeros M.A., Famoso A.N., Rebelo A.R., Singh N., Ma Q., Fei Z., Kochian L.V. (2017). ALUMINUM RESISTANCE TRANSCRIPTION FACTOR 1 (ART1) contributes to natural variation in aluminum resistance in diverse genetic backgrounds of rice (O. sativa). Plant Direct.

[28] Tyagi W., Yumnam J.S., Sen D., Rai M. (2020). Root transcriptome reveals efficient cell signaling and energy conservation key to aluminum toxicity tolerance in acidic soil adapted rice genotype. Sci. Rep..

[29] Chen Z.C., Yokosho K., Kashino M., Zhao F.J., Yamaji N., Ma J.F. (2013). Adaptation to acidic soil is achieved by increased numbers of cis-acting elements regulating ALMT1 expression in Holcus lanatus. Plant J..

