# Supplementary material for: Expression Level of Transcription Factor ART1 Is Responsible for Differential Aluminum Tolerance in Indica Rice
Source: Plants (Basel). 2021 Mar 26;10(4):634. doi: 10.3390/plants10040634 (PMC8066893; doi:10.3390/plants10040634)
Supplement: Supplementary file 1 [file plants-10-00634-s001.pdf]

Supplementary Materials:

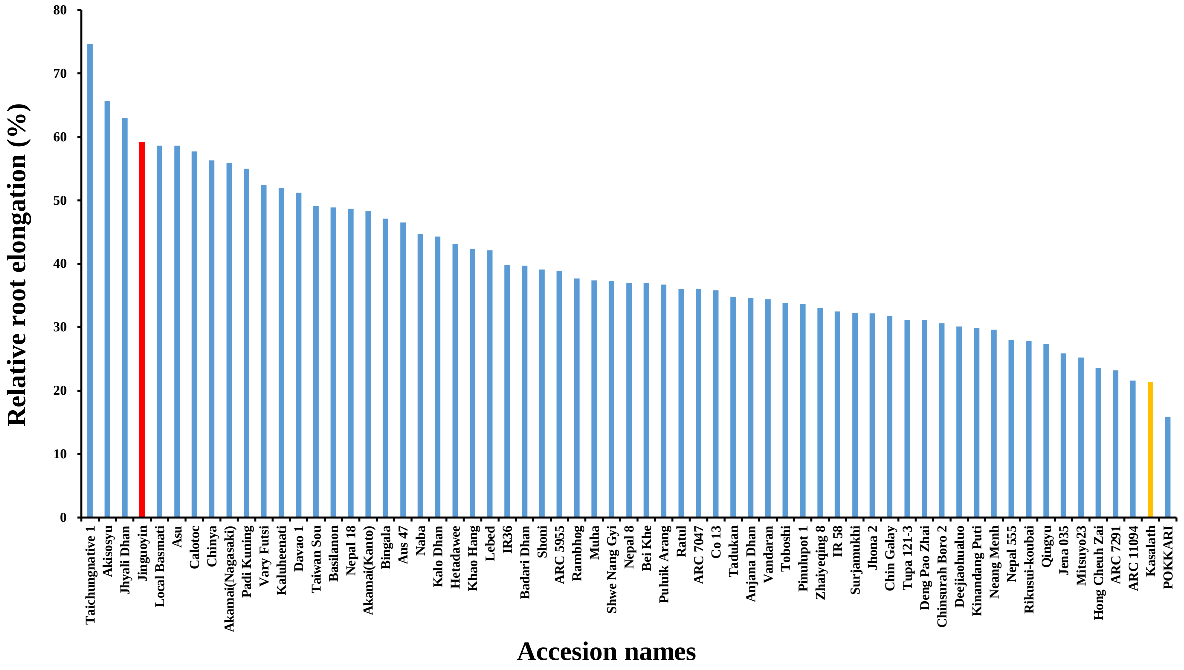

Supplementary Figure 1. Al tolerance of 65 *indica* rice varieties. Four-day-old seedlings were exposed to a 0.5 mM $CaCl_2$ (pH 4.5) solution containing 0 or 30 μM Al for 24 h. Relative root elongation was calculated as the following: root elongation with Al/root elongation without Al ╳ 100.

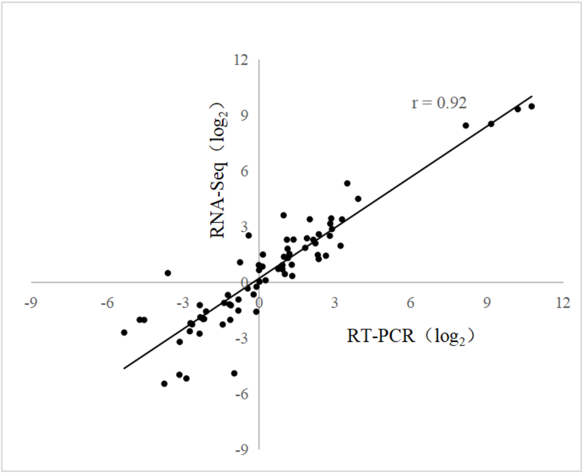

Supplementary Figure 2. Correlation of the gene expression ratio between RNA-seq data and quantitative real-time PCR (qRT-PCR) results. There were 17 genes randomly selected and subjected to qRT-PCR analysis. *Histone H3* was used as an internal standard. qRT-PCR data (fold change of the gene expression in response to Al) were plotted against data (fold change of the gene expression in response to Al) from RNA-seq. Both x and y-axes are shown in a log2 scale. r indicates the correlation coefficient.

(a)

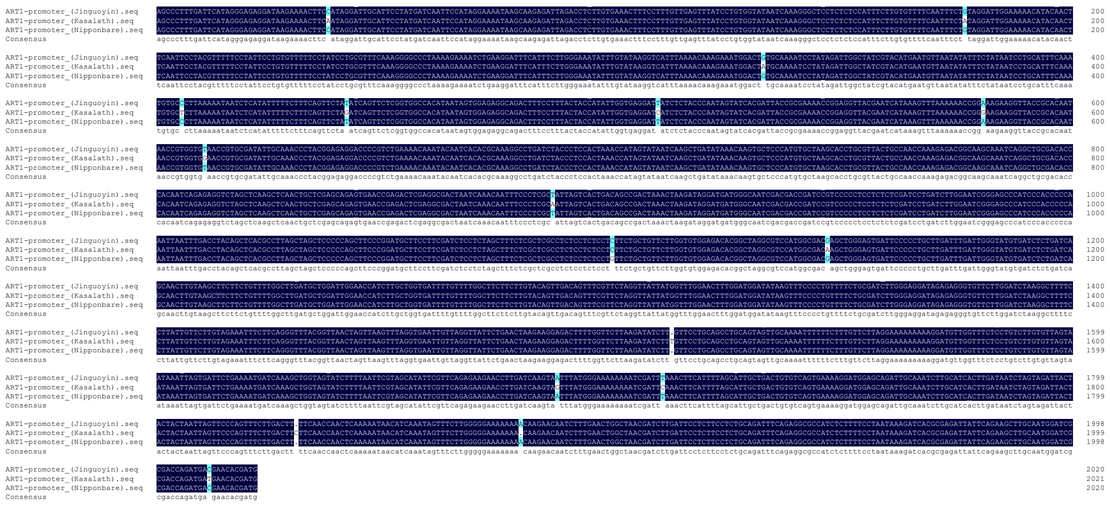

(b)

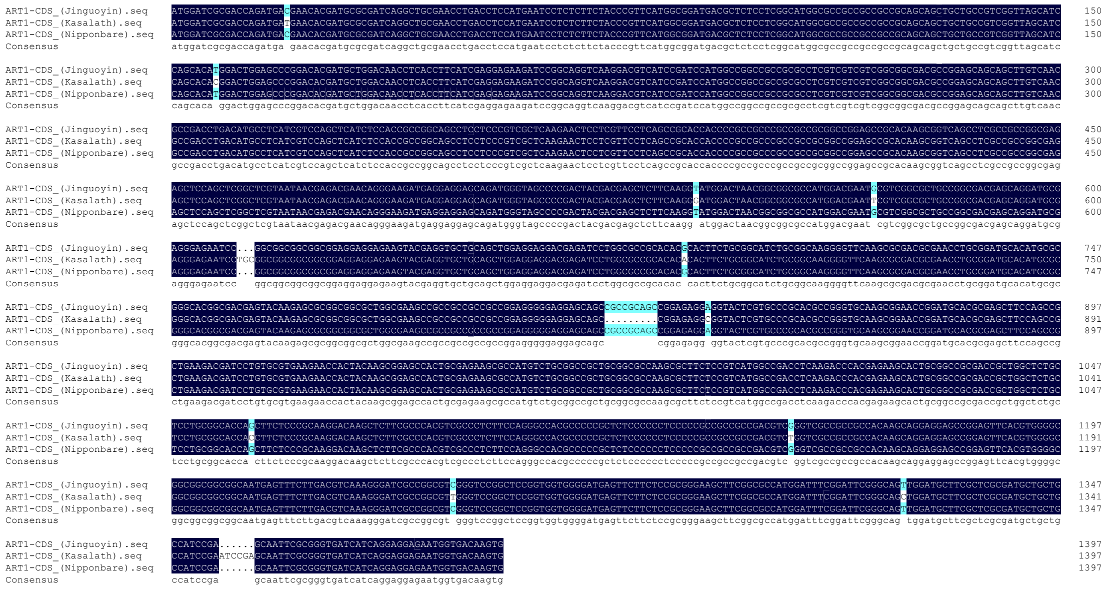

Supplementary Figure 3. Sequence comparison of the promoter (a) and coding sequence (CDS) regions (b) of *ART1* between Kasalath and Jinguoyin. The promoter and coding region sequences of Kasalath and Jinguoyin data were taken from the genome resequencing data, while the reference sequence of Nipponbare was taken from RAP-DB (https://rapdb.dna.affrc.go.jp/) and aligned by ClustalW.

Supplementary Table 1 Primer sequences used for investigating gene expression.

Gene	RAPDB ID	Forward primer	Reverse primer
Histone H3	Os06g0131001	GGTCAACTTGTTGATTCCCCTCT	AACCGCAAAATCCAAAGAACG
OsART2	Os04g0165200	GACAGATCACGTACGGGATTGA	CGAGCATGAAATGGGAACCTAC
OsCDT3	Os01g0178300	ATGTACAACCCTCCGGCGGC	TCAGCAGCAGCAGAGGCATTCG
OsMGT1	Os01g0869200	GCAAAGACTTGGAATCTCAGAC	CAGATGCGTACTTGTGTGAATC
OsFRDL4	Os01g0919100	CCATCTACATGAGCGTCAGAG	TCATTTGCGAAGAAACTTCCAC
OsNrat1	Os02g0131800	GAGGCCGTCTGCAGGAGAGG	GGAAGTATCTGCAAGCAGCTCTGATGC
OsALS1	Os03g0755100	ATTGTTTCAAGAGATGTTCGGC	CGAGCAACTACTCTCTCACTAG
OsSTAR2	Os05g0119000	ACCTCTTCATGGTCACCGTCG	CCTCAGCTTCTTCATCGTCACC
OsSTAR1	Os06g0695800	AGAACATCGAAGAGGCGATC	GGATCTGCTTCACGCTGT
OsFRDL2	Os10g0206800	TGATTCTTGTGGCAATTGTCAG	TTAAGAATCCTGCAAGCATTCG
OsART1	Os12g0170400	CAGTGCTTCTCGTGGGTCTT	CCTGTGCGTGAA GAACCACT
	Os09g0280500	CTGAGGAGGCTTGCACAGAA	CTGAAGGGCCAGAAGGAAGG
	Os06g0163000	AGAGGCAAACTCTGACGTGG	TGGACTGGATTAGGAGGCGA
	Os02g0528900	GTTTCAGGGCGTTGGCACTA	GCTCCTTTCTAAGCCTTCCACA
	Os06g0180300	CTCCGATTCTTGTGGCGTTT	GGTCCAGATCGTCTCCACG
	Os09g0266000	AGCTGCAGATCGTGTATGGG	TACAGCATGTTTGCAAGGCG
	Os06g0152600	CGCTTGCCGTGCTCATGTT	TACATGCGGTGGTGAGGCTTG
	Os02g0540700	ACCACCCACATCATGCCTTT	AGTCATTCTGCCCTTCCCTTG
	Os01g0935800	GCAGCATCTACCAAGACCCA	ATCCGTCACCTCGTTGTTCG
	Os04g0521100	CACTGGATCTTCTGGGTGGG	GACGCAGAAAGCCAGAAACC
	Os07g0551700	AGTTTGATCACTTCTAACCGCC	AGTGTGCGCTGGGTATATGT
	Os07g0689800	GTTTGAAGCGAAGCGTTGGT	TGCCTTCTGTGGGTCGTTTT
	Os10g0404300	ACTGTCTCGCATCGCATTCT	CGATCTCCGGCACTAGCAAT
	Os02g0264800	GATCGTTGTTTGGTGGTGCG	AAATGAACGAATCCAGCACGC
	Os06g0711900	GTGCATCGTCCTTGGTAGGC	GACCGGCGAAGAACAGCAAT
	Os01g0274500	GGAATTGAAGGGGCTCTGTTT	GGCAACATCAAAAGTACGCCA
